# Direct Synthesis of Chain-End Toluene Functionalized Hyperbranched Ethylene Oligomers

**DOI:** 10.3390/polym14153049

**Published:** 2022-07-28

**Authors:** Jianhai Chen, Zhengpeng Yan, Zhongyuan Li, Shengyu Dai

**Affiliations:** 1 Analysis and Testing Center, NERC Biomass of Changzhou University, Changzhou University, Changzhou 213164, China; jhchen@cczu.edu.cn; 2 School of Chemical and Environmental Engineering, Anhui Polytechnic University, Wuhu 241000, China; yan_zp1998@163.com; 3 Institutes of Physical Science and Information Technology, Key Laboratory of Structure and Functional Regulation of Hybrid Materials of Ministry of Education, Anhui University, Hefei 230601, China; 4 Anhui Laboratory of Molecule-Based Materials, Key Laboratory of Functional Molecular Solids, Ministry of Education, School of Chemistry and Materials Science, Anhui Normal University, Wuhu 241002, China; zhongyuanli@ahnu.edu.cn

**Keywords:** iminopyridine Ni(II) complexes, ethylene oligomerization, hyperbranched, toluene-end-functionalized

## Abstract

Chain-end functionalized polymers play an important role in the field of building complex macromolecular structures. In this study, we have synthesized and characterized four dibenzhydryl iminopyridine Ni(II) complexes bearing remote flexible substituents (Et and n-Bu) to provide hyperbranched ethylene oligomers in ethylene oligomerization with moderate to good activities. Most notably, toluene-end-functionalized hyperbranched ethylene oligomers were obtained under elevated temperature conditions and validated by NMR. The tandem catalysis of ethylene oligomerization and the subsequent Friedel–Crafts addition of the resulting unsaturated products to toluene molecules was proposed as the cause of the observed phenomenon.

## 1. Introduction

Chain-end functionalized polymers exhibit significant importance in the field of building complex macromolecular structures [1,2,3]. There are three main types of methods which have been reported to synthesize chain-end functionalized polymers. The first reported approach is that of living coordination polymerization of monomers, followed by in situ chain end reaction modification [4,5,6,7]. It is clear that such a method has inherent drawbacks, where the catalyst can only initiate one polymer chain per catalyst, thus limiting its efficiency and yield significantly. The second approach is the in situ one-pot method of chain-end functionalization by chain transfer [8,9,10,11,12,13,14,15,16]. Each catalyst is capable of generating a number of polymer chains. Due to the efficiency of this type of approach, it can play an important role in certain situations. For example, some early transition-metal catalysts are widely used in chain transfer chain-end functionalization reactions, which are capable of H-X σ-bond metathesis, such as an amine (H−NR_2_), borane (H−BR_2_). The final reported approach is chain-end functionalization of polymers by modification of unsaturated end groups of synthesized polymers, which is also a common approach. Though great success has been achieved with this strategy, there are several limitations which need to be overcome, such as difficulty in quantitatively converting terminal unsaturated bonds, due to the low concentration of terminal double bonds in polymers and the harshness of polymer solubility [17].

In recent years, an impressive effort has been devoted to exploring late-transition metal-catalyzed ethylene (co)oligomerization, which enables the synthesis of branched and even hyperbranched ethylene oligomers and co-oligomers [18,19,20,21,22,23,24,25]. Recently, many novel iminopyridine Ni(II) and Pd(II) complexes were designed for ethylene (co)oligomerization due to the unilateral axial steric structure of the iminopyridine ligands, which facilitates the chain transfer reaction during (co)oligomerization [26,27,28,29,30,31,32,33,34,35]. In this contribution, a series of rigid–flexible double-layer steric iminopyridine Ni(II) complexes, containing bulky diarylmethyl substituents with remote alkyl moieties, were synthesized and employed for ethylene oligomerization. Surprisingly, chain end toluene functionalized hyperbranched ethylene oligomers were prepared in situ in one pot.

## 2. Results and Discussions

### 2.1. Synthesis and Characterization of Iminopyridine Ni(II) Complexes

**Scheme 1:**
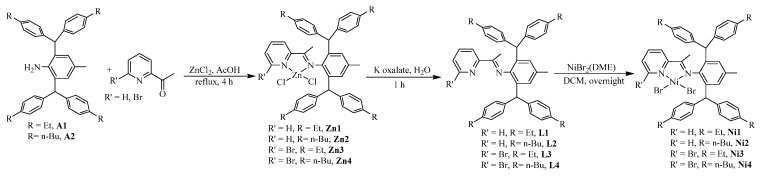
Synthesis of iminopyridine ligands and the corresponding nickel complexes.

**Figure 1:**
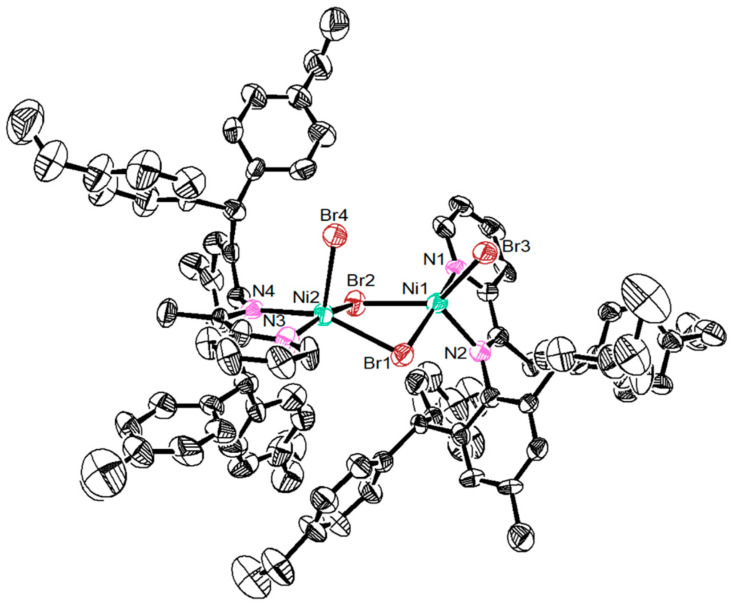
The single crystal structure of nickel complex **Ni1** (CCDC: 2080105; 30% probability level), H atoms and solvent molecules have been omitted for clarity.

Dibenzhydryl anilines **A1** and **A2** with remote flexible substituents (Et and n-Bu) were synthesized according to our previous work [30]. The iminopyridine ligands **L1**–**L4** were obtained from **A1** and **A2** with different 6-substituted 2-acetylpyridine by using the template-type method (Scheme 1) [30]. The ligands were obtained in good yields (60–72%) without chromatography and characterized by NMR (Figures S1–S8) and high-resolution mass spectra (Figures S9–S12). Then, the ligands reacted with 1.0 equiv. of NiBr_2_(DME) in dichloromethane at room temperature producing the corresponding Ni(II) complexes **Ni1**–**Ni4** in 75–85% yields (Scheme 1). The identity and purity of **Ni1**–**Ni4** were confirmed by MALDI-TOF MS (Figures S13–S16) and elemental analysis. A single crystal of **Ni1** was available from layering its CH_2_Cl_2_ solution with hexanes at ambient temperature (Figure 1). The complex **Ni1** was crystallized as a centrosymmetric dimer and each Ni(II) atom coordinated with two bridging bromine atoms and one iminopyridine ligand. One terminal bromine atom completed the square-pyramidal coordination sphere of the **Ni1** molecular structure. The dibenzhydryl groups deviated from the catalytic center, which might indicate that the complexes with dibenzhydryl substituents could not maintain an effective shielding on the catalytic center, leading to an easy chain transfer during the oligomerization process.

### 2.2. Ethylene Oligomerization

With 200 equiv. of Et_2_AlCl activated, the complexes **Ni1** and **Ni2** with H at the 6-position of the pyridine ring exhibited high activities (ca. 10^6^ g·mol^−1^·h^−1^), whereas the complexes **Ni3** and **Ni4** with Br at the same position performed moderate activities (ca. 10^5^ g·mol^−1^·h^−1^) of ethylene oligomerization (Table 1). All of the Ni(II) catalysts yielded low molecular weight (ca. 0.2–3.7 kg/mol) ethylene oligomers with high branching densities (76–94/1000 C) (Table 1). Interestingly, both the catalytic activity and molecular weight decreased as the reaction temperature increased. This was mainly attributed to the elevated temperatures enhancing chain transfer more than chain growth with the nickel catalysts and reducing the solubility of ethylene in toluene. Generally, complexes **Ni1**–**Ni2** with H at the 6-position of the pyridine ring exhibited higher activities than complexes **Ni3**–**Ni4** with Br, as well as yielding higher molecular weight ethylene oligomers, which was mainly due to the presence of the Br atom at the 6-position of the pyridine ring hindering the coordination and insertion of ethylene in the oligomerization process [32]. The slowing down of chain growth with little effect on chain transfer directly leads to a decrease of molecular weight. Compared to **Ni1** with an ethyl group, **Ni2** with a long chain butyl group showed lower activities and produced higher molecular weight ethylene oligomers at 30–70 °C (entries 1–3 vs. 4–6, Table 1). These results indicated that the long chain butyl groups were helpful to improve the molecular weight of the resulting oligomers at the expense of activity. The latter was mainly attributed to the fact that the long chain alkyl substituents with large steric hindrance facilitated the impediment of chain transfer reactions while, in the meantime, discouraging the coordination and insertion of ethylene.

**Figure 2:**
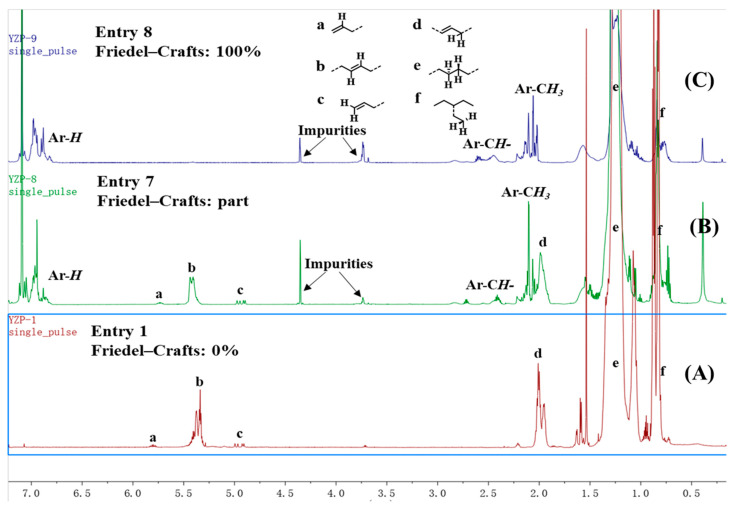
^1^H NMR spectrum (C_6_D_6_) of representative oligomer samples from Table 1, entries 1 (**A**), 7 (**B**) and 8 (**C**).

**Scheme 2:**
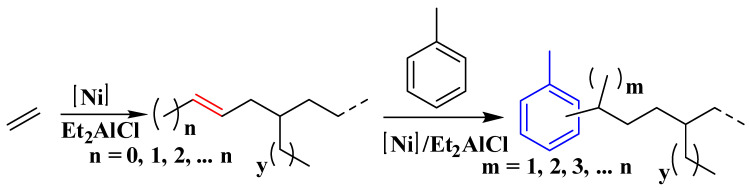
Mechanism of the tandem catalysis of ethylene oligomerization and the subsequent Friedel–Crafts addition to the toluene molecule.

**Figure 3:**
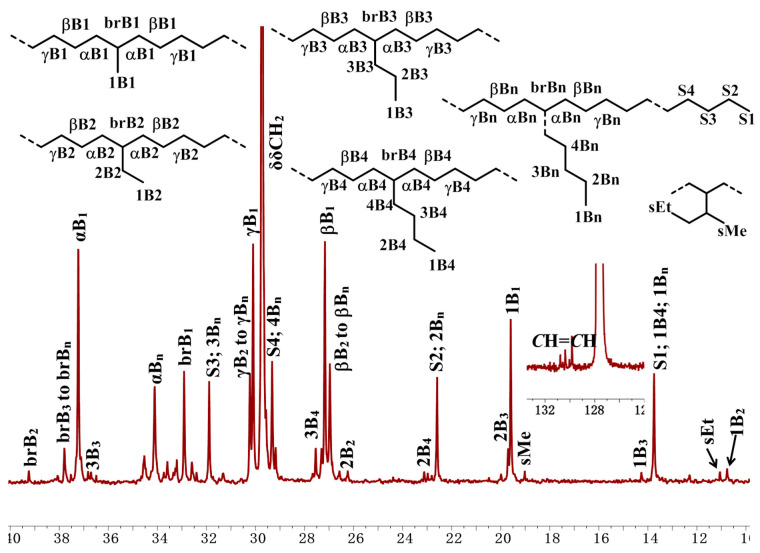
Detailed analysis of ^13^C NMR spectrum (C_6_D_6_) of the hyperbranched oligomer sample from entry 2, Table 1. Assignments are labeled by reference [36,37,38].

NMR spectra were utilized to analyze the microstructure of the produced ethylene oligomers. Interestingly, in the ^1^H NMR spectra of the ethylene oligomers formed at 30 and 50 °C in toluene with complexes **Ni1** and **Ni2** (Table 1, entries 1, 2, 4, and 5), internal olefinic (major, CH=CH, 5.48–5.30 ppm) or vinylic (minor, CH_2_=CH, 5.03 ppm and 4.95 ppm) proton signals were observed as the unsaturated chain end group (Figure 2A). However, the ethylene oligomers obtained at 30 °C in toluene with complexes **Ni3**–**Ni4** showed not only internal olefinic (major, CH=CH, 5.48–5.30 ppm) or vinylic (minor, CH2=CH, 5.03 ppm and 4.95 ppm) proton signals but also the aromatic proton resonances (6.80–7.22 ppm) (Figure 2B). Further raising the reaction temperature to 50–70 °C with **Ni1**–**Ni2** (70 °C) and **Ni3**–**Ni4** (50 and 70 °C) led to complete disappearance of olefinic proton signals and aromatic proton resonances (6.80–7.22 ppm) were detected exclusively as the chain ends (Figure 2C), which suggested the formation of toluene-end-functionalized branched ethylene oligomers. It implied that the process might undergo a tandem catalysis of ethylene oligomerization and the subsequent Friedel–Crafts addition of the resulting unsaturated ethylene oligomers to solvent molecules (Scheme 2). More importantly, the ratio of toluene-end-functionalized ethylene oligomers could be facilely controlled by catalyst structure and reaction temperature. The microstructure of a typical ethylene oligomer (Table 1, entry 2) was verified by the ^13^C NMR analysis (Figure 3) [36,37,38]. The ^13^C NMR spectrum confirmed the presence of branches of various chain lengths (B1, B2, B3 and Bn branches), olefinic carbons, and sec-butyl structures (Figure 3). Among them, terminal methyl group and methyl branches dominated all the branches, and hyperbranched structures were observed as well, as evidenced by the existence of sec-butyl groups [18]. The chain-end toluene functionalized hyperbranched ethylene oligomers could be used in lubricant and surfactant applications [18].

## 3. Conclusions

In summary, we have synthesized and characterized four dibenzhydryl iminopyridine Ni(II) complexes bearing remote flexible substituents (Et and n-Bu). Complexes **Ni1**–**Ni2** with H at the 6-position of the pyridine ring performed high activities (ca. 10^6^ g·mol^−1^·h^−1^) while complexes **Ni3**–**Ni4** with Br at the 6-position of the pyridine showed moderate activities (ca. 10^5^ g·mol^−1^·h^−1^) of ethylene oligomerization. Highly branched (76–94/1000 C) ethylene oligomers with various molecular weight sizes were produced in the above catalytic system. Most notably, toluene-end-functionalized hyperbranched ethylene oligomers, validated by NMR, were obtained under elevated temperature conditions. A plausible mechanism was also demonstrated, which underwent tandem catalysis of ethylene oligomerization and the subsequent Friedel–Crafts addition of the resulting unsaturated ethylene oligomers to toluene.

## 4. Experimental Sections

### 4.1. General Considerations

All chemicals were commercially sourced, except those having synthesis as described. All experiments were carried out under a dry nitrogen atmosphere using standard Schlenk techniques or in a glove-box. Deuterated solvents used for NMR were dried and distilled prior to use. The ^1^H and ^13^C NMR spectra were recorded by a JNM-ECZ600R or JNM-ECZ400R spectrometer at ambient temperature unless otherwise stated. The chemical shifts of the ^1^H and ^13^C NMR spectra were referenced to the residual solvent, with coupling constants in Hz. Mass spectra were obtained by the Analytical Center of Anhui University. Elemental analysis was performed by the Analytical Center of Anhui University. X-ray Diffraction data were collected at 293(2) K on a Bruker Smart CCD area detector with graphite-mono-chromated Mo Kα radiation (λ = 0.71073 Å). The molecular weight and the molecular weight distribution of the polymers were determined by gel permeation chromatography (GPC) equipped with two linear Styragel columns (HR2 and HR4) at 40 °C, using THF as a solvent and calibrated with polystyrene standards. THF was employed as the eluent at a flow rate of 1.0 mL/min.

### 4.2. Procedure for the Synthesis of Ligands **L1**–**L4**

Anilines **A1**–**A2** were synthesized according to our previous work [30]. The ligands **L1**–**L4** were prepared as follows: ZnCl_2_ (0.34 g, 2.5 mmol) and 2-acetylpyridine (3.0 mmol), were suspended in glacial acetic acid (5 mL). Anilines (2 mmol) were then added, and the reaction mixture was refluxed under stirring for 4 h. The solution was allowed to cool to room temperature, and a bright yellow solid precipitated. The solid was separated by filtration and washed with acetic acid (3 × 5 mL) and diethyl ether (5 × 5 mL) to remove the remaining acetic acid. Drying under vacuum gave a bright yellow and poorly soluble solid. Then, the zinc was removed from the zinc diimine complex. The product of the previous step was suspended in methylene chloride (30 mL), and a solution of potassium oxalate (0.41 g, 2.2 mmol) in water (5 mL) was added. The reaction mixture was stirred vigorously for 1 h. The two phases were separated, and the organic layer was washed with water (3 × 20 mL) and dried with MgSO_4_. After filtration, the solvent was removed under vacuum to obtain the product as a yellow powder and dried under high vacuum. The ligands **L1**–**L2** were also known [30].

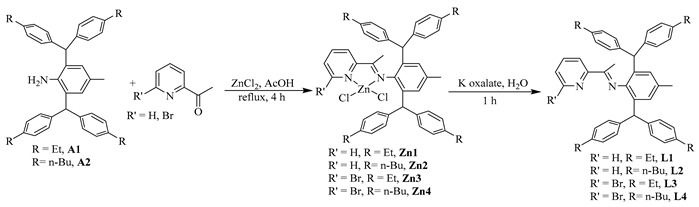



**L1** (0.92 g, 70%). ^1^H NMR (600 MHz, CDCl_3_) δ 8.61 (d, *J* = 3.9 Hz, 1H, Ar-*H*), 8.10 (d, *J* = 8.1 Hz, 1H, Ar-*H*), 7.80–7.66 (m, 1H, Ar-*H*), 7.42–7.30 (m, 1H, Ar-*H*), 7.08 (d, *J* = 8.0 Hz, 4H, Ar-*H*), 7.02 (d, *J* = 7.9 Hz, 4H, Ar-*H*), 6.98 (d, *J* = 8.0 Hz, 4H, Ar-*H*), 6.95 (d, *J* = 8.0 Hz, 4H, Ar-*H*), 6.76 (s, 2H, Ar-*H*), 5.25 (s, 2H, C*H*Ar_2_), 2.62 (dq, *J* = 23.1, 7.6 Hz, 8H, C*H*_2_CH_3_), 2.22 (s, 3H, Ar-C*H*_3_), 1.24 (t, *J* = 7.6 Hz, 6H, CH_2_C*H*_3_), 1.21 (t, *J* = 7.6 Hz, 6H, CH_2_C*H*_3_), 1.13 (s, 3H, Ar-C(C*H*_3_)=N). ^13^C NMR (151 MHz, CDCl_3_) δ 169.43 (*C*=N), 156.33, 148.50, 146.10, 141.94, 141.67, 141.46, 140.19, 136.10, 132.53, 131.46, 129.79, 129.43, 128.52, 127.75, 127.51, 124.58, 121.50, 51.34 (*C*HAr_2_), 28.51 (*C*H_2_CH_3_), 28.49 (*C*H_2_CH_3_), 21.46 (Ar-*C*H_3_), 17.03 (Ar-C(*C*H_3_)=N), 15.67 (CH_2_*C*H_3_), 15.63 (CH_2_*C*H_3_). MALDI-TOF-MS (*m*/*z*): calcd for C_48_H_50_N_2_: 654.4000, Found, 654.4003, [M + H]^+^.

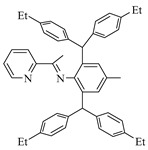



**L2** (1.01 g, 66%). ^1^H NMR (600 MHz, CDCl_3_) δ 8.59 (d, *J* = 4.1 Hz, 1H, Ar-*H*), 8.05 (d, *J* = 7.8 Hz, 1H, Ar-*H*), 7.70 (t, *J* = 7.2 Hz, 1H, Ar-*H*), 7.39–7.29 (m, 1H, Ar-*H*), 7.03 (d, *J* = 7.9 Hz, 4H, Ar-*H*), 6.94 (dt, *J* = 11.9, 8.0 Hz, 12H, Ar-*H*), 6.71 (s, 2H, Ar-*H*), 5.21 (s, 2H, C*H*Ar_2_), 2.65–2.46 (m, 8H, C*H*_2_CH_2_CH_2_CH_3_), 2.19 (s, 3H, Ar-C*H*_3_), 1.68–1.48 (m, 8H, CH_2_C*H*_2_CH_2_CH_3_), 1.45–1.20 (m, 8H, CH_2_CH_2_C*H*_2_CH_3_), 1.08 (s, 3H, Ar-C(C*H*_3_)=N), 0.92 (q, *J* = 7.1 Hz, 12H, CH_2_CH_2_CH_2_C*H*_3_). ^13^C NMR (151 MHz, CDCl_3_) δ 169.39 (*C*=N), 156.32, 148.49, 146.12, 141.41, 140.56, 140.32, 140.14, 136.03, 132.56, 131.40, 129.72, 129.35, 128.50, 128.30, 128.04, 124.53, 121.47, 51.35 (*C*HAr_2_), 35.34 (*C*H_2_CH_2_CH_2_CH_3_), 35.27 (*C*H_2_CH_2_CH_2_CH_3_), 33.72 (CH_2_*C*H_2_CH_2_CH_3_), 33.70 (CH_2_*C*H_2_CH_2_CH_3_), 22.52 (CH_2_CH_2_*C*H_2_CH_3_), 22.41 (CH_2_CH_2_*C*H_2_CH_3_), 21.45 (Ar-*C*H_3_), 16.99 (Ar-C(*C*H_3_)=N), 14.09 (CH_2_CH_2_CH_2_*C*H_3_), 14.05 (CH_2_CH_2_CH_2_*C*H_3_). MALDI-TOF-MS (*m*/*z*): calcd for C_55_H_66_N_2_: 766.5200, Found, 766.5245, [M + H]^+^.

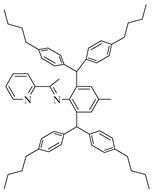



**L3** (1.06 g, 72%). ^1^H NMR (600 MHz, CDCl_3_) δ 7.97 (d, *J* = 7.6 Hz, 1H, Ar-*H*), 7.55 (t, *J* = 7.7 Hz, 1H, Ar-*H*), 7.53–7.49 (m, 1H, Ar-*H*), 7.06 (d, *J* = 8.0 Hz, 4H, Ar-*H*), 7.02 (d, *J* = 8.0 Hz, 4H, Ar-*H*), 6.95 (d, *J* = 8.0 Hz, 4H, Ar-*H*), 6.91 (d, *J* = 8.1 Hz, 4H, Ar-*H*), 6.72 (s, 2H, Ar-*H*), 5.16 (s, 2H, C*H*Ar_2_), 2.66–2.54 (m, 8H, C*H*_2_CH_3_), 2.19 (s, 3H, Ar-C*H*_3_), 1.26–1.19 (m, 12H, CH_2_C*H*_3_), 1.12 (s, 3H, Ar-C(C*H*_3_)=N). ^13^C NMR (151 MHz, CDCl_3_) δ 168.54 (*C*=N), 157.40, 145.77, 142.06, 141.73, 141.17, 140.75, 140.04, 138.42, 132.44, 131.73, 129.74, 129.39, 128.95, 128.49, 127.82, 127.53, 120.12, 51.45 (*C*HAr_2_), 28.52 (*C*H_2_CH_3_), 28.50 (*C*H_2_CH_3_), 21.44 (Ar-*C*H_3_), 17.01 (Ar-C(*C*H_3_)=N), 15.72 (CH_2_*C*H_3_), 15.62 (CH_2_*C*H_3_). APCI-MS (*m*/*z*): calcd for C_48_H_49_BrN_2_: 735.3092, Found, 735.3110, [M + H]^+^.

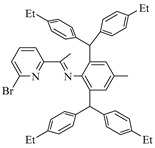



**L4** (1.02 g, 60%). ^1^H NMR (600 MHz, CDCl_3_) δ 8.05 (dd, *J* = 7.5, 0.9 Hz, 1H, Ar-*H*), 7.60–7.50 (m, 2H, Ar-*H*), 7.10 (d, *J* = 8.1 Hz, 4H, Ar-*H*), 7.06 (d, *J* = 8.0 Hz, 4H, Ar-*H*), 7.01 (d, *J* = 8.0 Hz, 4H, Ar-*H*), 6.97 (d, *J* = 7.9 Hz, 4H, Ar-*H*), 6.78 (s, 2H, Ar-*H*), 5.23 (s, 2H, C*H*Ar_2_), 2.63 (dd, *J* = 16.3, 8.8 Hz, 8H, C*H*_2_CH_2_CH_2_CH_3_), 2.24 (s, 3H, Ar-C*H*_3_), 1.70–1.58 (m, 8H, CH_2_C*H*_2_CH_2_CH_3_), 1.45–1.35 (m, 8H, CH_2_CH_2_C*H*_2_CH_3_), 1.14 (s, 3H, Ar-C(C*H*_3_)=N), 0.99 (t, *J* = 7.3 Hz, 12H, CH_2_CH_2_CH_2_C*H*_3_). ^13^C NMR (151 MHz, CDCl_3_) δ 168.64 (*C*=N), 157.42, 145.89, 141.19, 140.82, 140.74, 140.45, 140.06, 138.43, 132.57, 131.74, 129.76, 129.40, 129.00, 128.55, 128.49, 128.14, 120.12, 51.58 (*C*HAr_2_), 35.39 (*C*H_2_CH_2_CH_2_CH_3_), 35.34 (*C*H_2_CH_2_CH_2_CH_3_), 33.79 (CH_2_*C*H_2_CH_2_CH_3_), 22.58 (CH_2_CH_2_*C*H_2_CH_3_), 22.43 (CH_2_CH_2_*C*H_2_CH_3_), 21.49 (Ar-*C*H_3_), 16.99 (Ar-C(*C*H_3_)=N), 14.16 (CH_2_CH_2_CH_2_*C*H_3_), 14.14 (CH_2_CH_2_CH_2_*C*H_3_). APCI-MS (*m*/*z*): calcd for C_56_H_65_BrN_2_: 847.4344, Found, 847.4363, [M + H]^+^.

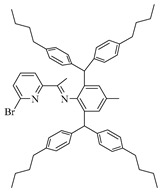



### 4.3. Procedure for the Synthesis of Nickel Complexes **Ni1**–**Ni4**

Complexes **Ni1**–**Ni4** were synthesized by the reaction of 1 equiv. of NiBr_2_(DME) with the corresponding ligands in methylene chloride. The corresponding ligand (0.2 mmol) was added in 5 mL of methylene chloride in a Schlenk tube under a nitrogen atmosphere. NiBr_2_(DME) (0.2 mmol, 62 mg) was added to the above solution. The resulting mixture was stirred at room temperature overnight. The solvent was evaporated under reduced pressure to afford a solid. The product was washed with 4 × 5 mL hexane and dried under vacuum. A single crystal could be obtained by diffusion from layering hexanes on to the CH_2_Cl_2_ solution at room temperature.

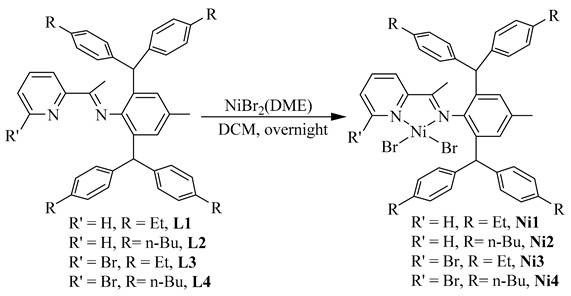



**Ni1** (0.14 g, 80%), Elem. Anal. Calcd for C_48_H_50_Br_2_N_2_Ni: C, 66.01; H, 5.77; N, 3.21. Found: C, 66.21; H, 5.59; N, 3.11. MALDI-TOF-MS (*m*/*z*): calcd for C_48_H_50_BrN_2_Ni: 791.2511, Found, 791.2515, [M − Br]^+^.

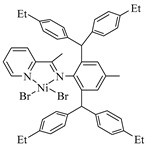



**Ni2** (0.17 g, 85%). Elem. Anal. Calcd for C_56_H_66_Br_2_N_2_Ni: C, 68.24; H, 6.75; N, 2.84. Found: C, 68.35; H, 6.95; N, 3.05. MALDI-TOF-MS (*m*/*z*): calcd for C_56_H_66_BrN_2_Ni: 903.3763, Found, 903.3737, [M − Br]^+^.

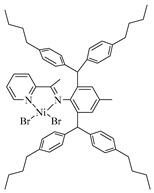



**Ni3** (0.15 g, 80%). Elem. Anal. Calcd for C_48_H_49_Br_3_N_2_Ni: C, 60.54; H, 5.19; N, 2.94. Found: C, 60.35; H, 5.27; N, 3.01. MALDI-TOF-MS (*m*/*z*): calcd for C_48_H_49_Br_2_N_2_Ni: 869.1616, Found, 869.1609, [M − Br]^+^.

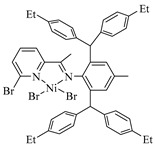



**Ni4** (0.16 g, 75%). Elem. Anal. Calcd for C_56_H_65_Br_3_N_2_Ni: C, 63.18; H, 6.15; N, 2.63. Found: C, 63.35; H, 6.28; N, 2.72. MALDI-TOF-MS (*m*/*z*): calcd for C_56_H_65_Br_2_N_2_Ni: 981.2868, Found, 981.2879, [M − Br]^+^.

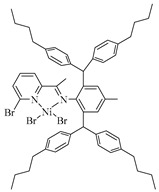



### 4.4. A General Procedure for the Ethylene Oligomerization Using Ni Complexes

In a typical experiment, a pressure glass reactor with a 350 mL thick wall, connected with a high-pressure gas line, was first dried at 90 °C under vacuum for at least 1 h. The reactor was then adjusted to the desired oligomerization temperature. Then, 20 mL of toluene and the desired amount Et_2_AlCl was added to the reactor under N_2_ atmosphere, and the desired amount of catalyst in 1 mL of CH_2_Cl_2_ was injected into the oligomerization system via syringe. With rapid stirring, the reactor was pressurized and maintained at 6 atm of ethylene. After 60 min, the pressure reactor was vented and the ethylene oligomers were obtained under vacuum.

## Figures and Tables

**Table 1:** Effect of Catalysts and Temperatures on Ethylene Oligomerization.

Ent.	Precat.	*T*/°C	Yield/g	Act. *^b^*	*M*_n_ *^c^*	*M*_w_/*M*_n_*^c^*	*B ^d^*	F–C *^e^*
1	Ni1	30	3.54	1.77	3.6	1.42	78	0%
2	Ni1	50	3.10	1.55	3.1	1.38	84	0%
3	Ni1	70	2.98	1.49	2.8	1.28	93	100%
4	Ni2	30	3.02	1.51	3.6	1.45	78	0%
5	Ni2	50	2.81	1.41	3.7	1.37	79	0%
6	Ni2	70	2.31	1.16	3.0	1.31	94	100%
7	Ni3	30	1.00	0.50	1.3	1.04	81	Part
8	Ni3	50	0.65	0.33	0.8	1.04	76	100%
9	Ni3	70	0.47	0.24	0.2 *^f^*	- *^f^*	- *^f^*	100%
10	Ni4	30	0.45	0.23	1.6	1.05	76	Part
11	Ni4	50	0.43	0.22	1.2	1.03	78	100%
12	Ni4	70	0.30	0.15	0.2 *^f^*	- *^f^*	- *^f^*	100%

*^a^* General conditions: complexes (2 μmol), Et_2_AlCl (200 equiv.), 1 mL CH_2_Cl_2_, 20 mL toluene, time = 1.0 h, ethylene = 6 atm. *^b^* Activity (Act.) = 10^6^ g/(mol Ni·h). *^c^* Molecular weights (kg mol^−1^) determined by GPC in THF at 40 °C vs. polystyrene standards. *^d^* brs = Number of branches per 1000 C, as determined by ^1^H NMR spectroscopy. *^e^* Conversion of the Friedel–Crafts reaction, as determined by ^1^H NMR spectroscopy. *^f^* Molecular weight below the detection limit of GPC, GC-MS detection of molecular weight around 0.2 kg/mol.

## Data Availability

All the data can be obtained in the supplementary materials or by contacting the corresponding author.

## Supplementary Materials

The following are available online at https://www.mdpi.com/article/10.3390/polym14153049/s1. NMR, GPC and GC-MS curves of oligomers samples, single crystal data of **Ni1**.
