# Supplementary material for: Direct Synthesis of Chain-End Toluene Functionalized Hyperbranched Ethylene Oligomers
Source: Polymers (Basel). 2022 Jul 28;14(15):3049. doi: 10.3390/polym14153049 (PMC9370379; doi:10.3390/polym14153049)
Supplement: Supplementary file 1 [file polymers-14-03049-s001.zip › polymers-1824004-supplementary.pdf]

Direct Synthesis of Chain-end Toluene Functionalized Hyperbranched Ethylene Oligomers

Jianhai Chen†[a], Zhengpeng Yan†[b, c], Zhong-Yuan Li[d], Shengyu Dai[b, c]*

[a]*Analysis and Testing Center, NERC Biomass of Changzhou University, School of Pertrochemical Engineering, Changzhou University, Changzhou 213164, China.*
[b]*School of Chemical and Environmental Engineering, Anhui Polytechnic University, Wuhu, Anhui 241000, China.*
[c]*Institutes of Physical Science and Information Technology, Key Laboratory of Structure and Functional Regulation of Hybrid Materials of Ministry of Education, Anhui University, Hefei, Anhui, 230601, China.*
[d]*Anhui Laboratory of Molecule-Based Materials; Key Laboratory of Functional Molecular Solids, Ministry of Education; School of Chemistry and Materials Science, Anhui Normal University, Wuhu, 241002, China.*

†The first two authors are equal first authors.

*To whom correspondence should be addressed. daiyu@ustc.edu.cn (Shengyu Dai, +86/13955189794).

1. Spectra Data

1.1 ^{1}H and ^{13}C NMR of the Synthetic Compounds.

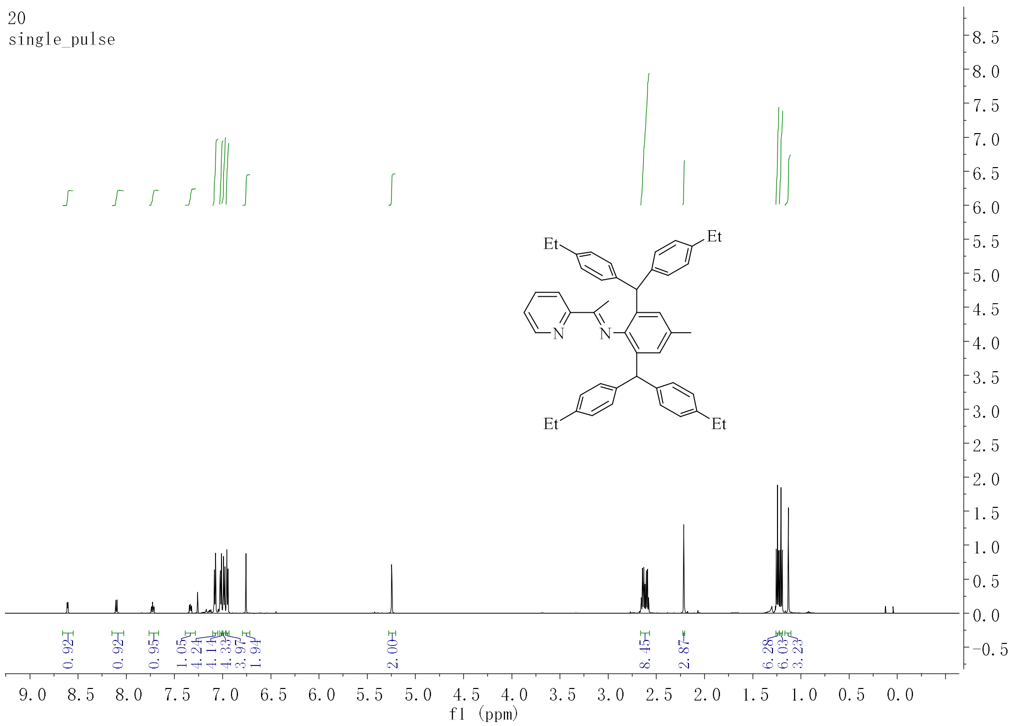

Figure S1. ^{1}H NMR spectrum of **L1** in $CDCl_3$.

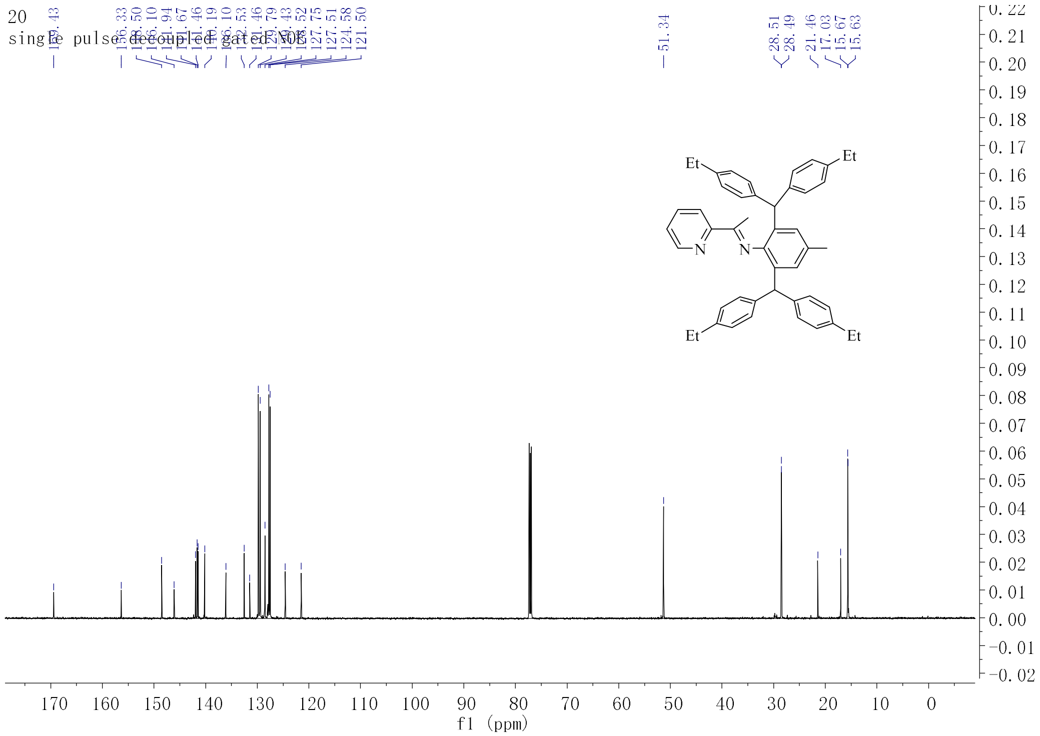

Figure S2. ^{13}C NMR spectrum of **L1** in $CDCl_3$.

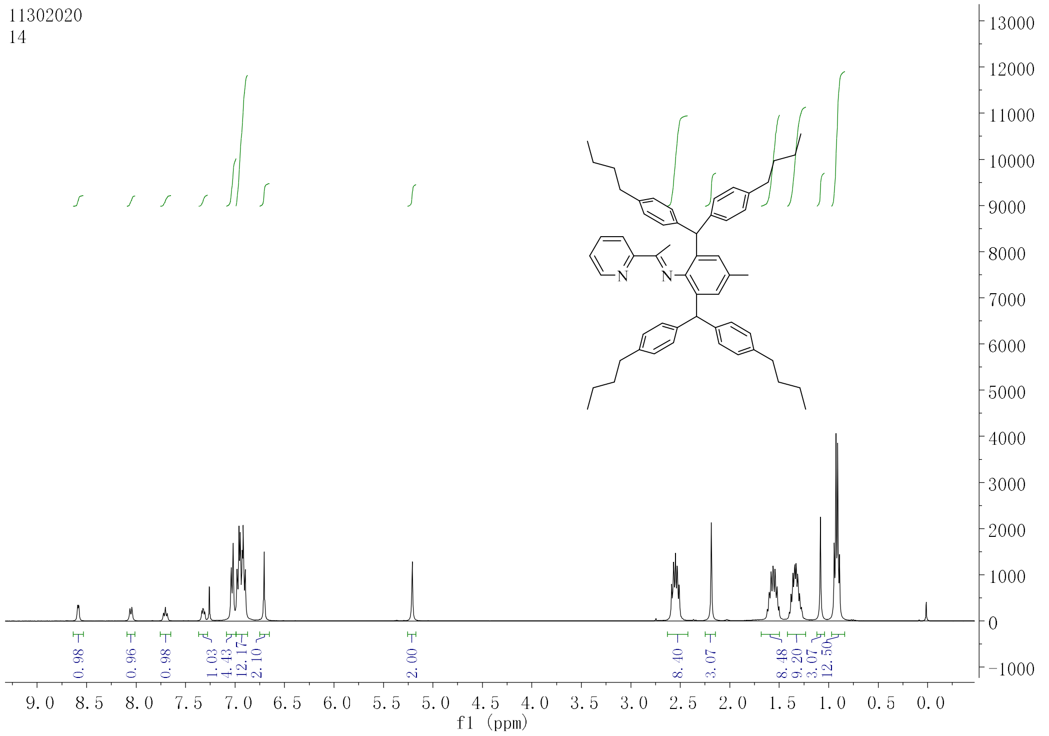

Figure S3. ^{1}H NMR spectrum of **L2** in $CDCl_3$.

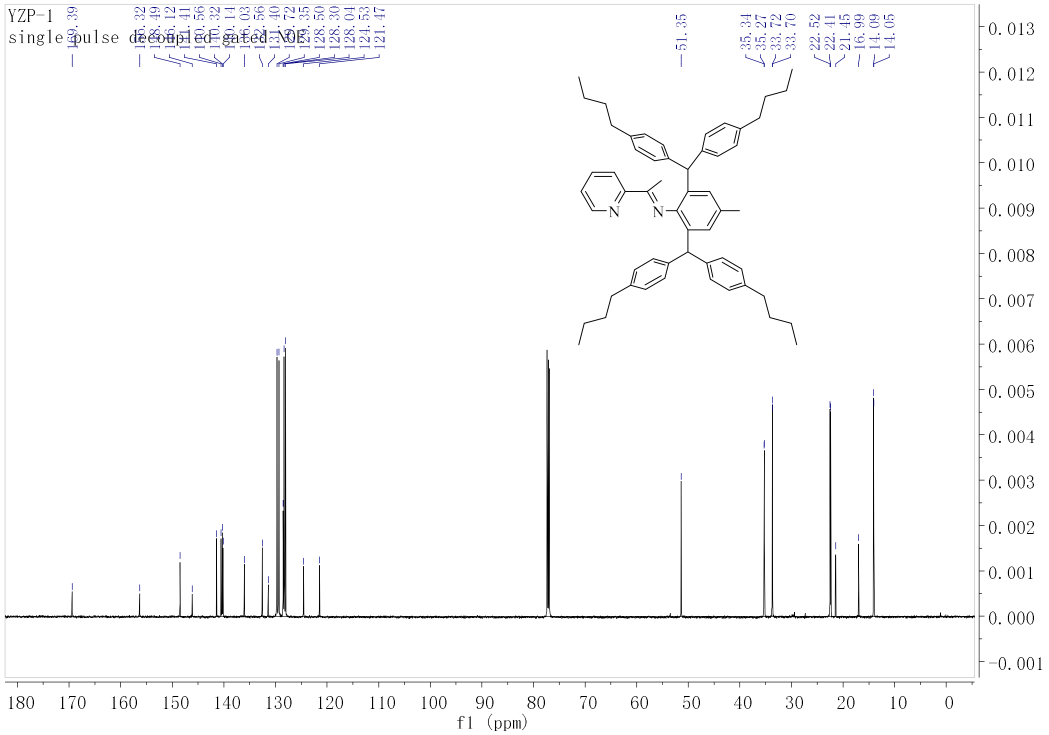

Figure S4. ^{13}C NMR spectrum of **L2** in $CDCl_3$.

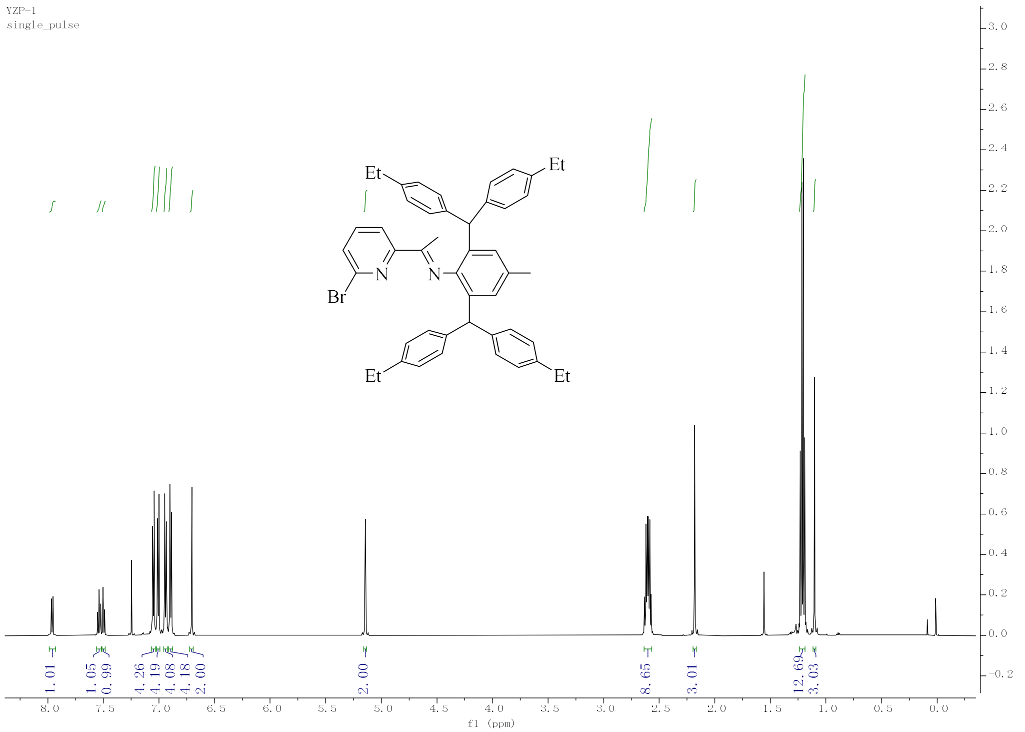

Figure S5. ^{1}H NMR spectrum of **L3** in $CDCl_3$.

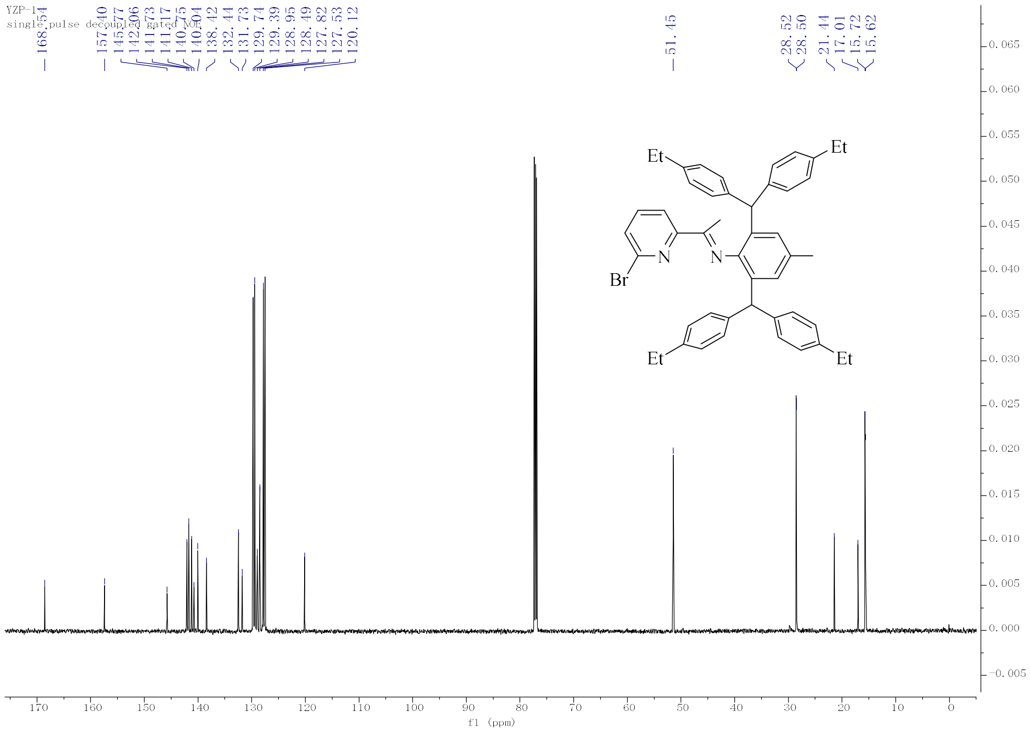

Figure S6. ^{13}C NMR spectrum of **L3** in $CDCl_3$.

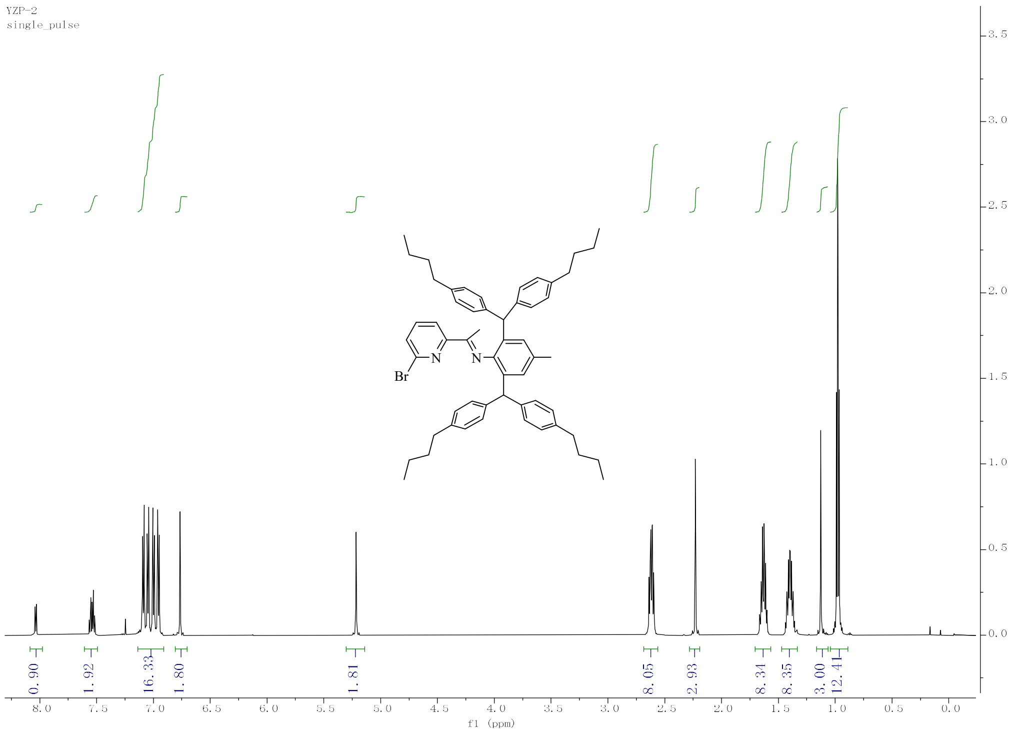

Figure S7. ^{1}H NMR spectrum of **L4** in $CDCl_3$.

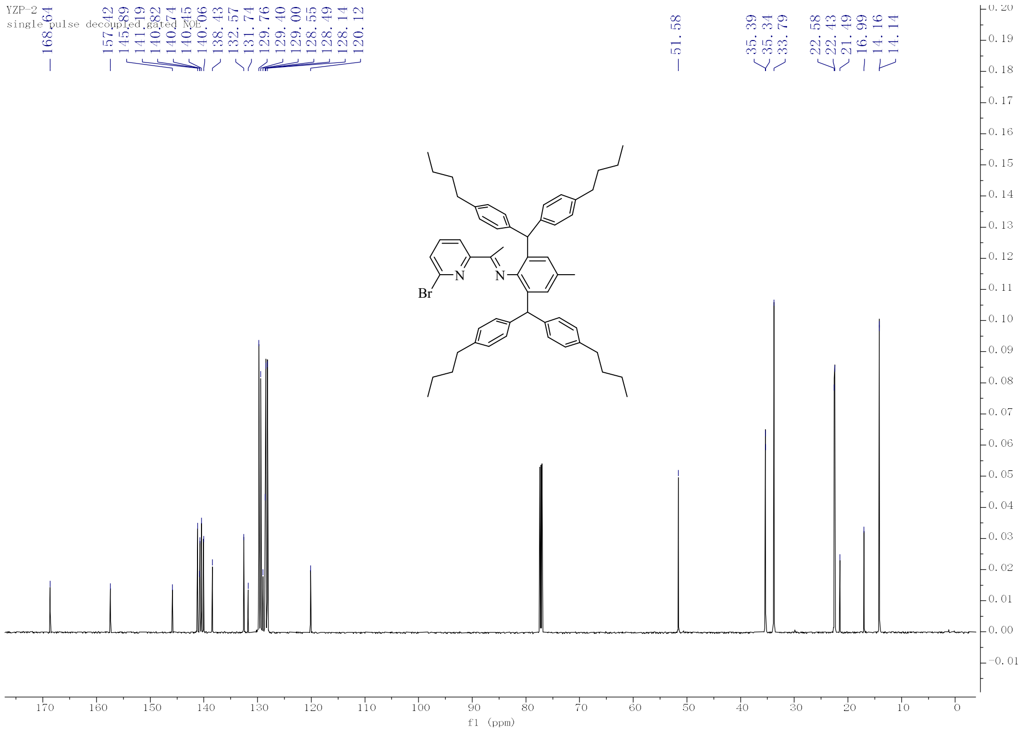

Figure S8. ^{13}C NMR spectrum of **L4** in $CDCl_3$.

1.2 MS of L1-L4.

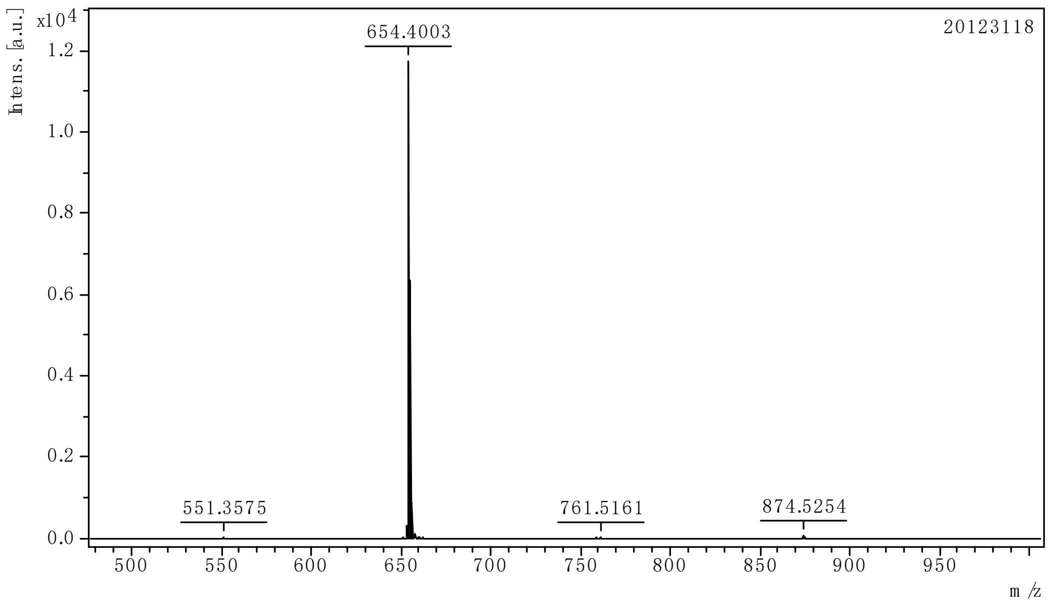

Figure S9. MALDI-TOF-MS of **L1**.

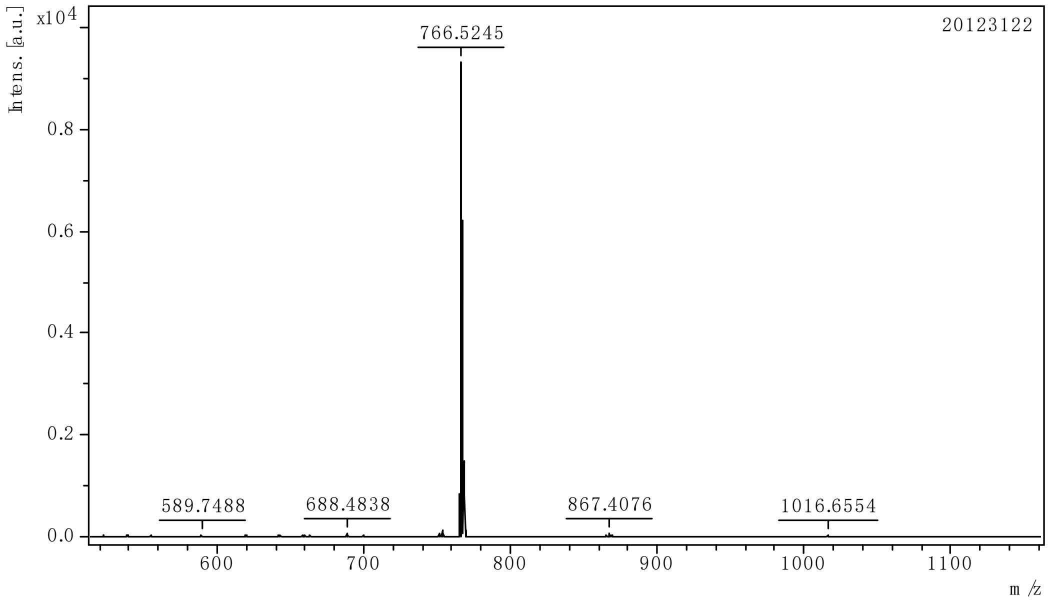

Figure S10. MALDI-TOF-MS of **L2**.

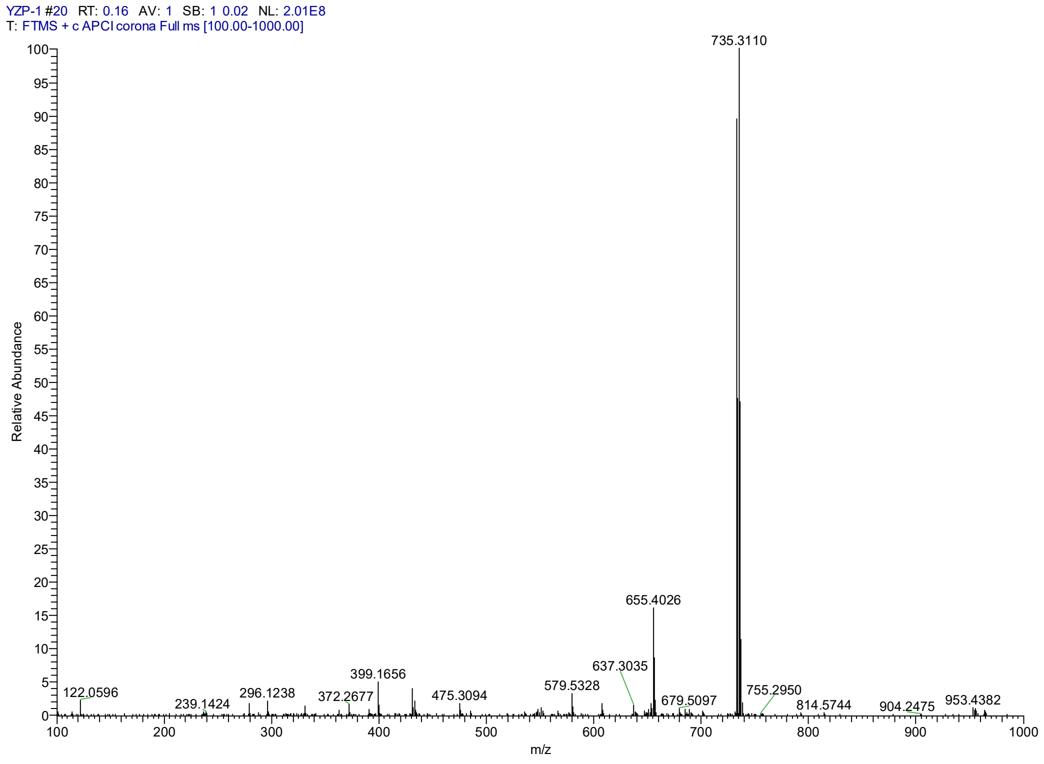

Figure S11. APCI-MS of **L3**.

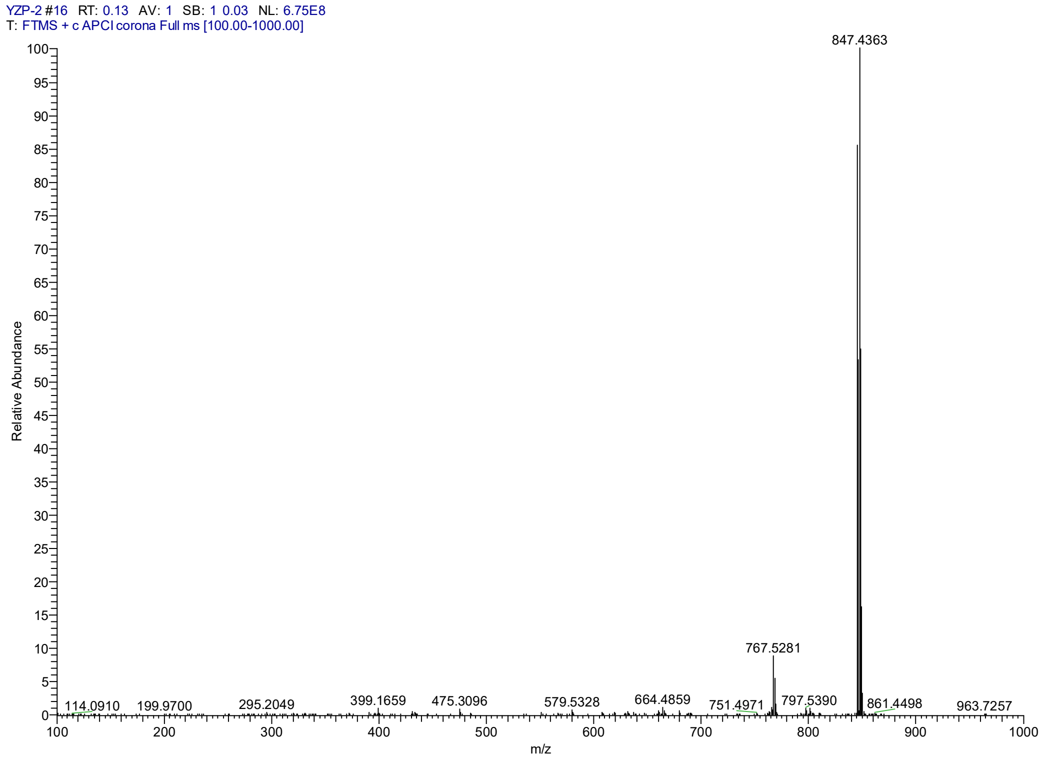

Figure S12. APCI-MS of **L4**.

1.3 MS of Complexes Ni1-Ni4

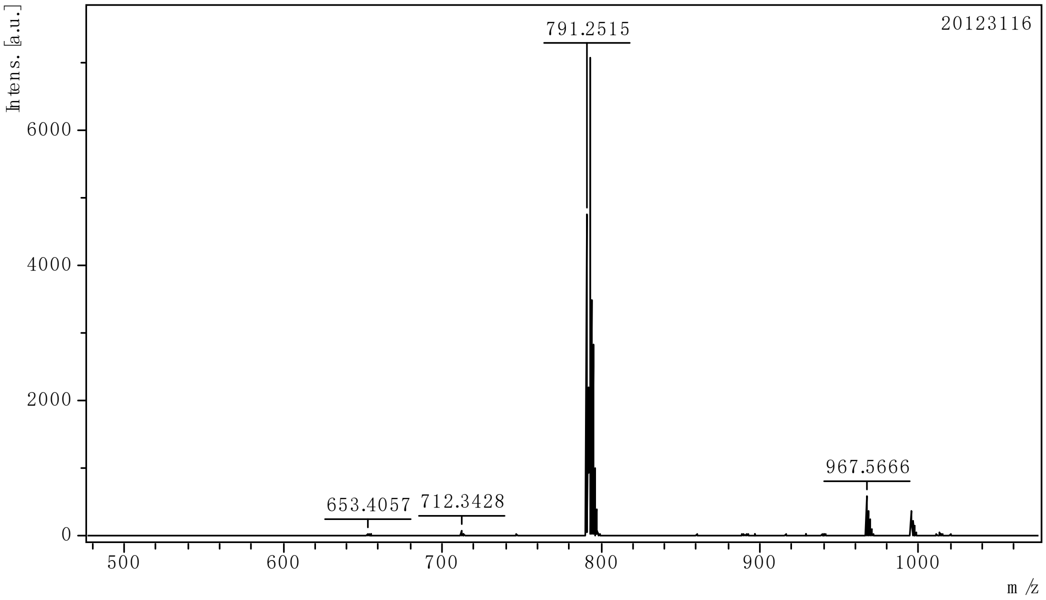

Figure S13. MALDI-TOF-MS of **Ni1**.

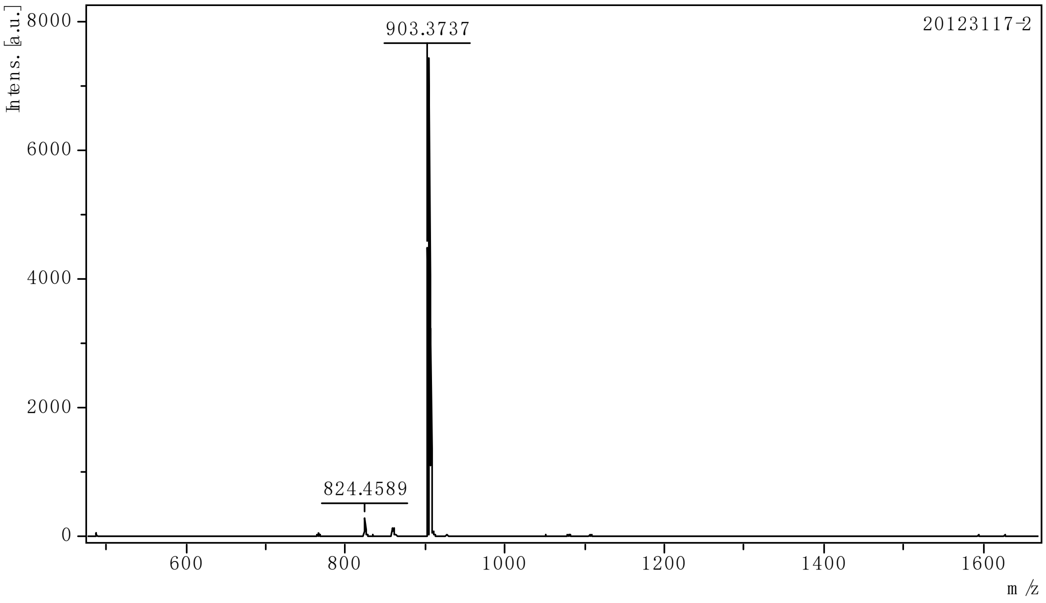

Figure S14. MALDI-TOF-MS of **Ni2**.

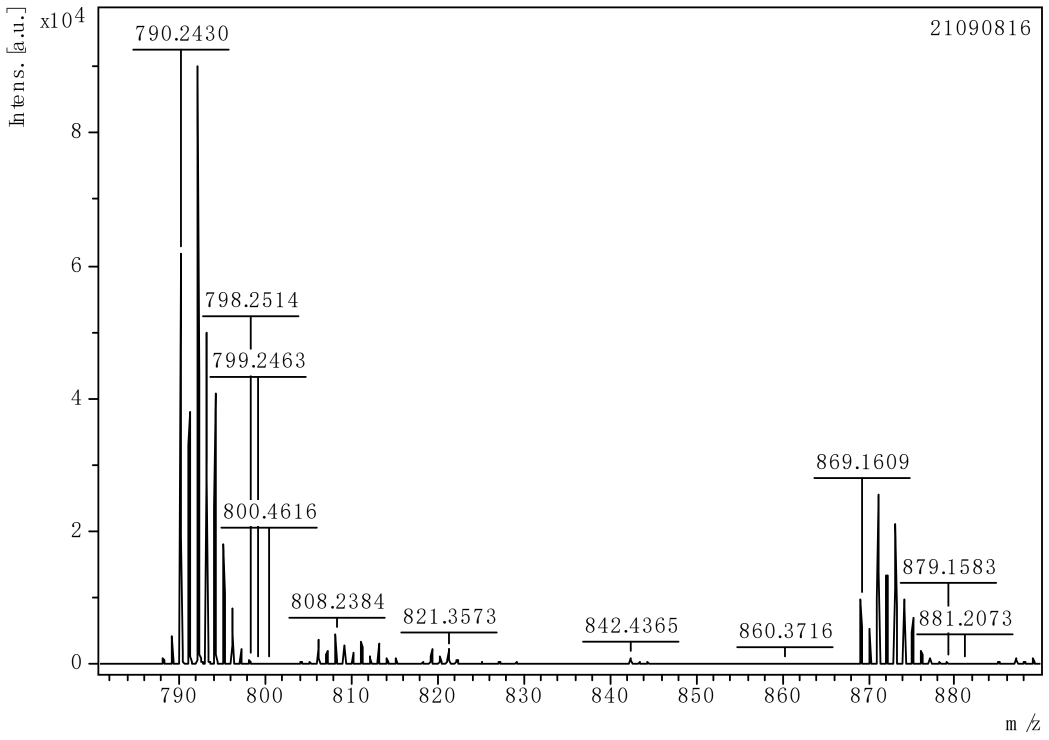

Figure S15. MALDI-TOF-MS of **Ni3**.

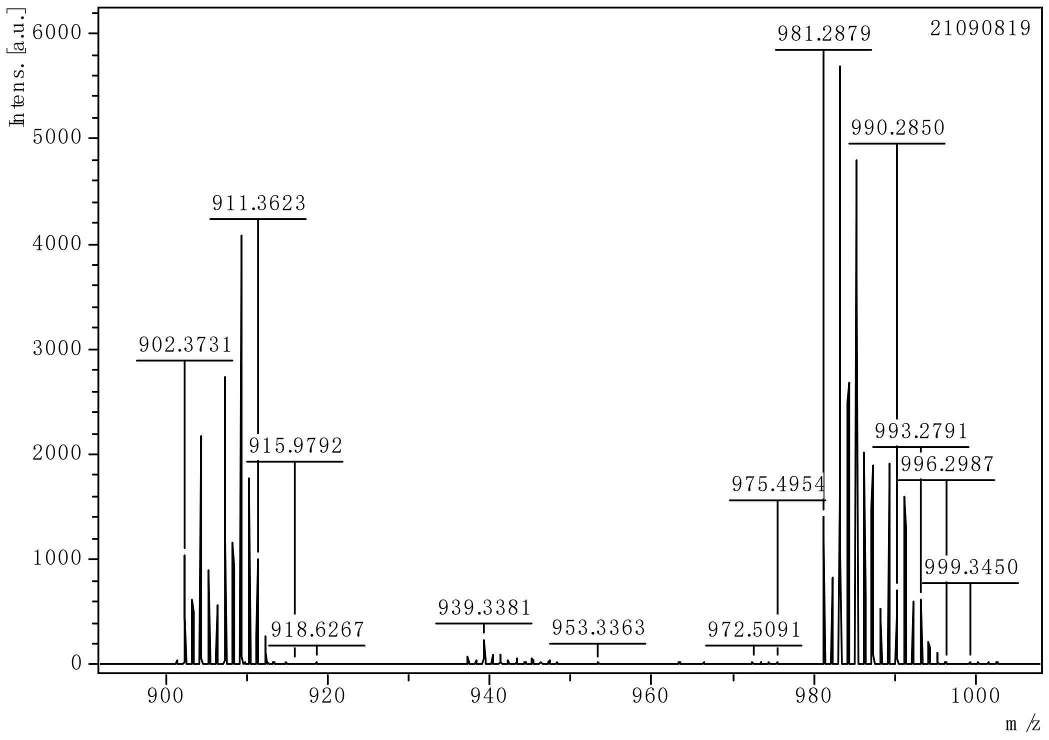

Figure S16. MALDI-TOF-MS of **Ni4**.

1.4 ^{1}H and ^{13}C NMR of Representative Ethylene Oligomers.

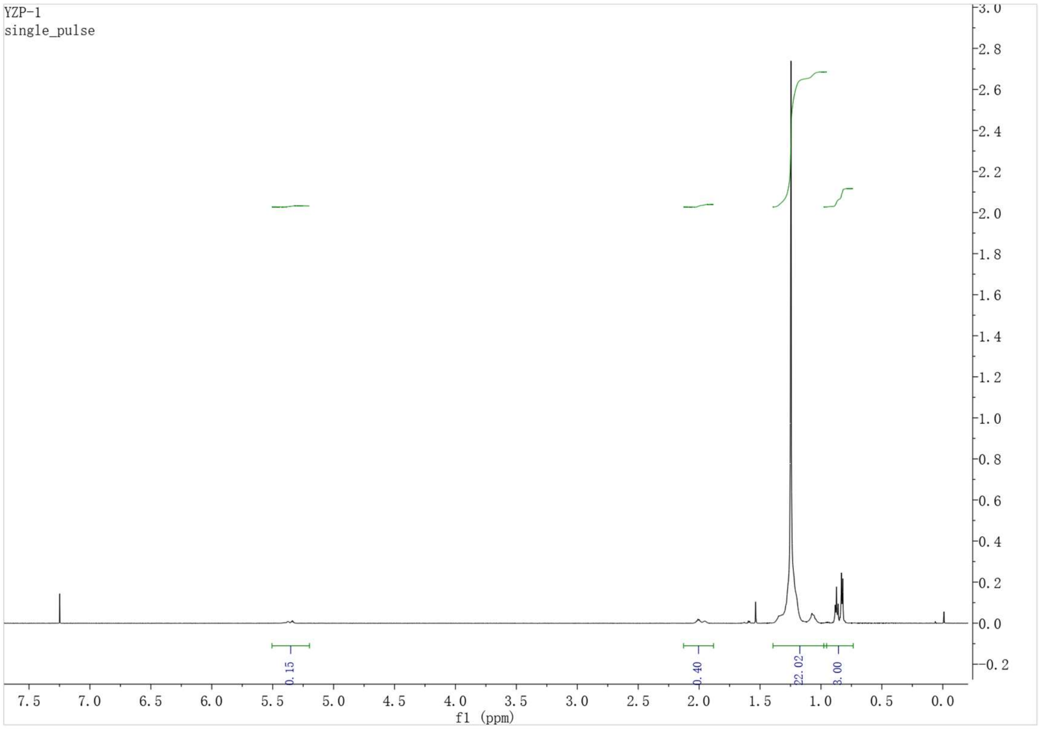

Figure S17. ^{1}H NMR spectrum of the polymer from table 1, entry 1 ($CDCl_3$, 20 °C).

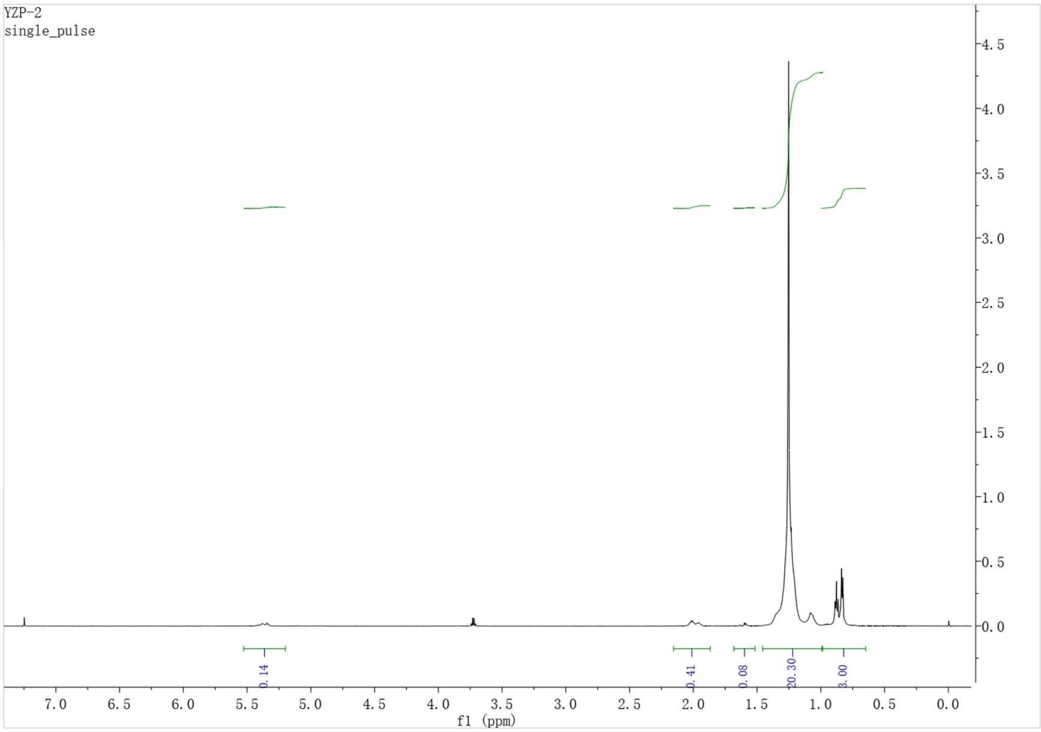

Figure S18. ^{1}H NMR spectrum of the polymer from table 1, entry 2 ($CDCl_3$, 20 °C).

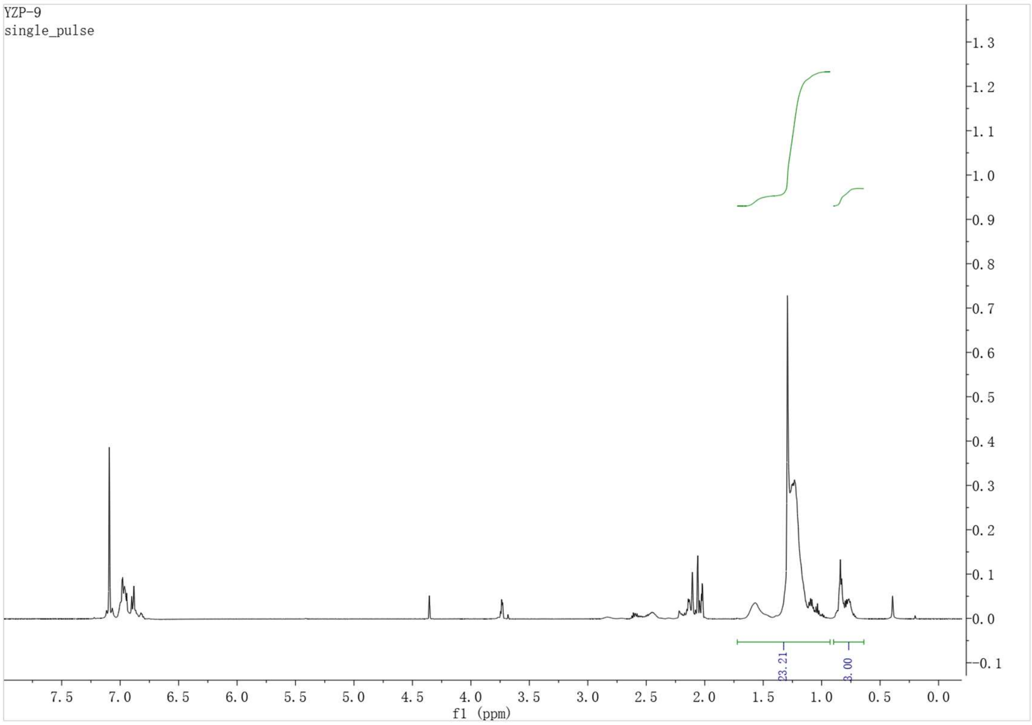

Figure S19. ^{1}H NMR spectrum of the polymer from table 1, entry 8 ($CDCl_3$, 20 °C).

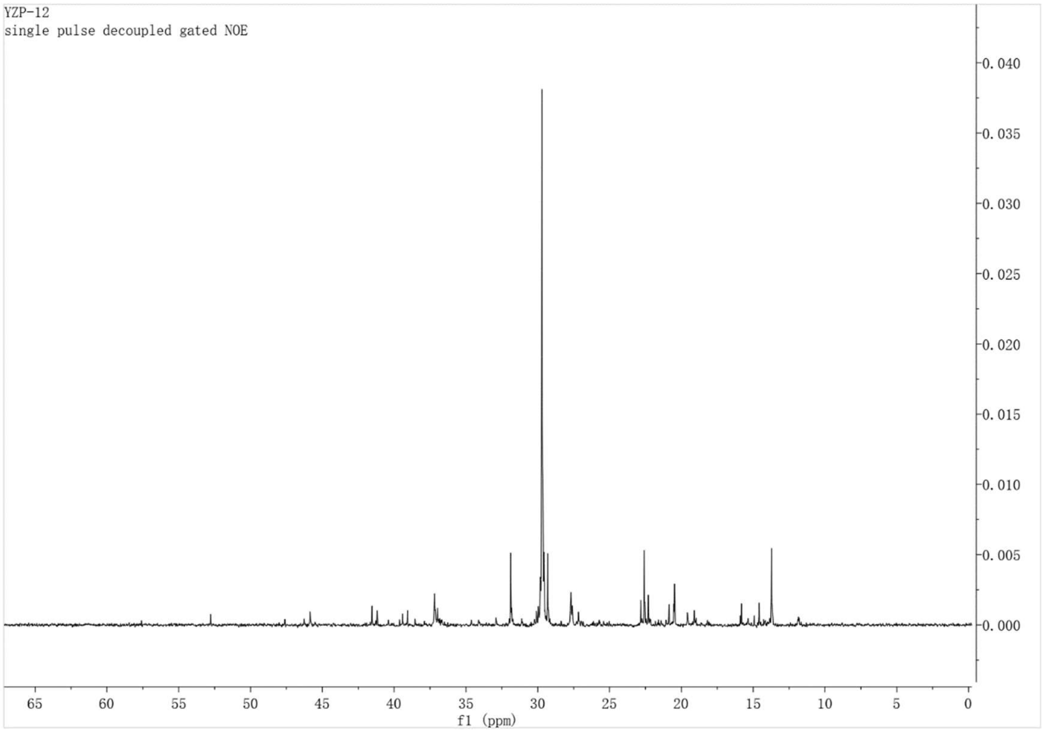

Figure S20. ^{13}C NMR spectrum of the polymer from table 1, entry 11 ($CDCl_3$, 20 °C).

1.5 GPC and GC-MS of Representative Ethylene Oligomers.

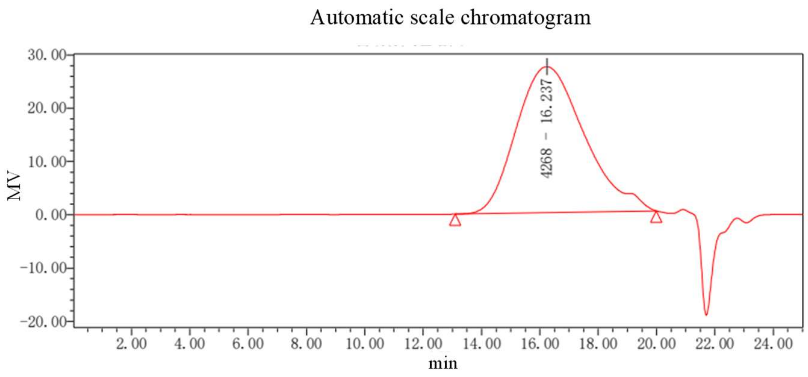

GPC Results

	Distribution name	Mn (Dalton)	Mw (Dalton)	MP	Mz (Dalton)	Mz+1 (Dalton)	Mv	PDI	Mw Mark1	Mw Mark2
1		3574	5058	4268	7039	9278		1.415168		

Figure S21. GPC of the oligomer from table 1, entry 1.

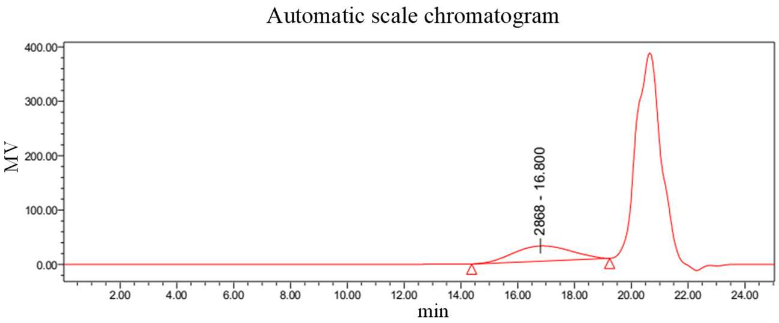

GPC Results

	Distribution name	Mn (Dalton)	Mw (Dalton)	MP	Mz (Dalton)	Mz+1 (Dalton)	Mv	PDI	Mw Mark1	Mw Mark2
1		2797	3575	2868	4584	5685		1.278126		

Figure S22. GPC of the oligomer from table 1, entry 3.

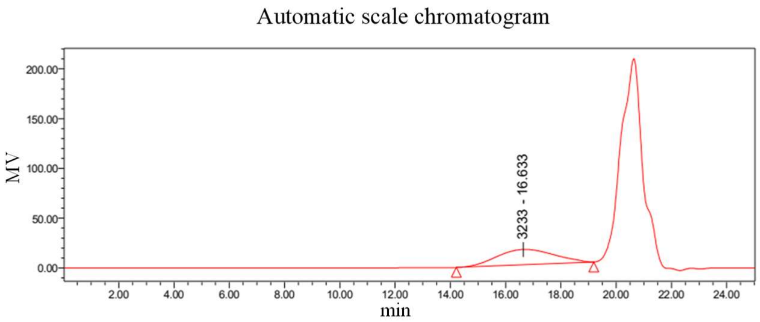

GPC Results

	Distribution name	Mn (Dalton)	Mw (Dalton)	MP	Mz (Dalton)	Mz+1 (Dalton)	Mv	PDI	Mw Mark1	Mw Mark2
1		2986	3901	3233	5074	6338		1.306409		

Figure S23. GPC of the polymer from table 1, entry 6.

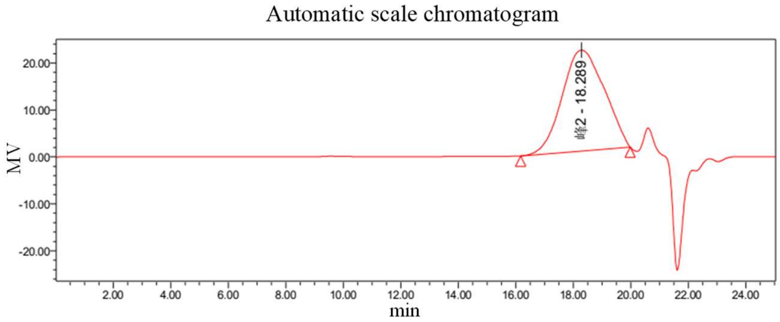

GPC Results

	Distribution name	Mn (Dalton)	Mw (Dalton)	MP	Mz (Dalton)	Mz+1 (Dalton)	Mv	PDI	Mw Mark1	Mw Mark2
1		1642	1720		1819	1944		1.047323		

Figure S24. GPC of the polymer from table 1, entry 10.

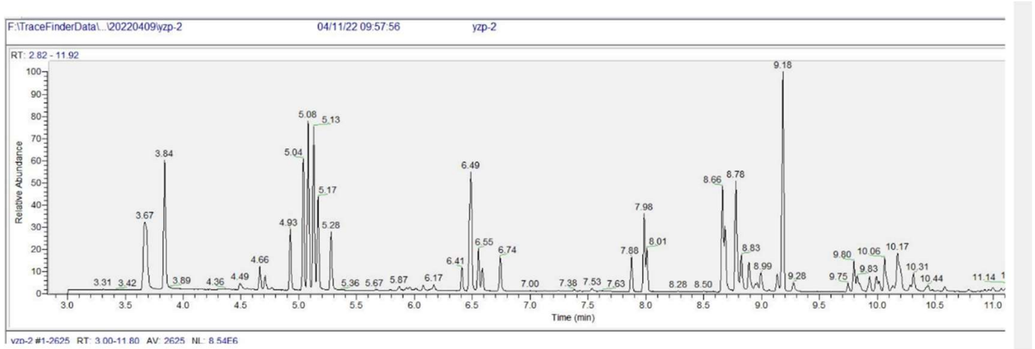

Figure S25. GC-MS of the polymer from table 1, entry 12.

2. References

[1] Yan, Z.; Li, S.; Dai, S. Synthesis and characterization of hyperbranched polar functionalized olefin oligomers. *Chin. J. Synth. Chem.* **2021**, *29*, 1033–1044.

3. X-ray Crystallography.

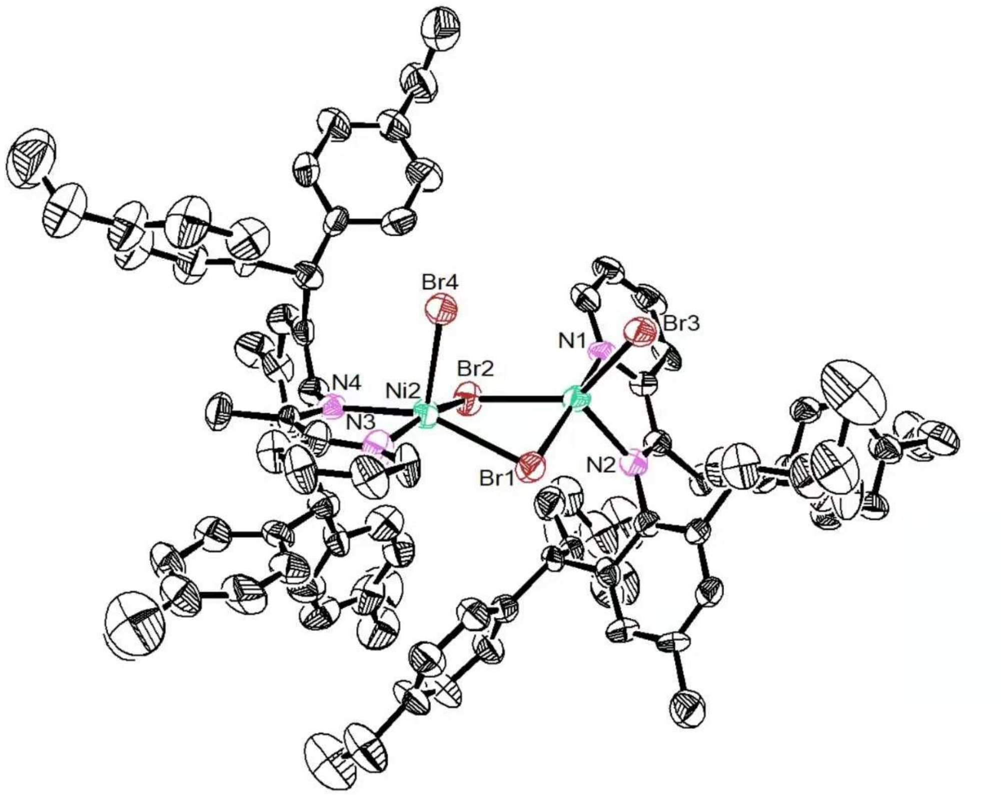

Table S1 Crystal data and structure refinement for Ni1.

Identification code	**Ni1**
Empirical formula	C97.5 H103 Br4 N4 Ni2
Formula weight	1874.25
Temperature/K	298(2)
Crystal system	Triclinic
Space group	P-1
a/Å	16.0191(14)
b/Å	18.2202(15)
c/Å	19.2910(17)
α/°	98.562(3)
β/°	91.765(2)
γ/°	111.897(5)
Volume/Å^3	5143.1(8)

Z	2
ρ_{calc} g/cm^3	1.210
μ/mm^{-1}	2.039
F(000)	1926
Crystal size/mm^3	0.34 x 0.21 x 0.17
Radiation	MoKα (λ = 0.71073)
2Θ range for data collection/°	1.82 to 25.02
Index ranges	-19<=h<=12, -21<=k<=21, -22<=l<=22
Reflections collected	25536
Independent reflections	17798 [R(int) = 0.0751]
Data/restraints/parameters	17798 / 745 / 1032
Goodness-of-fit on F^2	1.093
Final R indexes [I>=2σ (I)]	R1 = 0.1009, wR2 = 0.2391
Final R indexes [all data]	R1 = 0.2156, wR2 = 0.2732
Largest diff. peak/hole / e Å^{-3}	1.206 and -1.626
